# Protein Turnover and Cellular Stress in Mildly and Severely Affected Muscles from Patients with Limb Girdle Muscular Dystrophy Type 2I

**DOI:** 10.1371/journal.pone.0066929

**Published:** 2013-06-28

**Authors:** Simon Hauerslev, Marie L. Sveen, John Vissing, Thomas O. Krag

**Affiliations:** Neuromuscular Research Unit, Department of Neurology, Rigshospitalet, University of Copenhagen, Copenhagen, Denmark; Institut de Myologie, France

## Abstract

Patients with Limb girdle muscular dystrophy type 2I (LGMD2I) are characterized by progressive muscle weakness and wasting primarily in the proximal muscles, while distal muscles often are spared. Our aim was to investigate if wasting could be caused by impaired regeneration in the proximal compared to distal muscles. Biopsies were simultaneously obtained from proximal and distal muscles of the same patients with LGMD2I (n = 4) and healthy subjects (n = 4). The level of past muscle regeneration was evaluated by counting internally nucleated fibers and determining actively regenerating fibers by using the developmental markers embryonic myosin heavy chain (eMHC) and neural cell adhesion molecule (NCAM) and also assessing satellite cell activation status by myogenin positivity. Severe muscle histopathology was occasionally observed in the proximal muscles of patients with LGMD2I whereas distal muscles were always relatively spared. No difference was found in the regeneration markers internally nucleated fibers, actively regenerating fibers or activation status of satellite cells between proximal and distal muscles. Protein turnover, both synthesis and breakdown, as well as cellular stress were highly increased in severely affected muscles compared to mildly affected muscles. Our results indicate that alterations in the protein turnover and myostatin levels could progressively impair the muscle mass maintenance and/or regeneration resulting in gradual muscular atrophy.

## Introduction

Limb girdle muscular dystrophy type 2I (LGMD2I), the most common form of recessive LGMD in Scandinavia, is caused by mutations in the gene encoding the fukutin-related protein (FKRP). α-Dystroglycan (α-DG) is a component of the dystrophin-glycoprotein complex (DGC) and contains multiple sites for O-linked glycosylation facilitated by FKRP and other glycosyltransferases [1]. Proper O-glycosylation of α-DG is crucial for its interaction with the extracellular laminin-α2 and agrin in muscle [2]. α-DG hypoglycosylation affects these interactions and the disruption of the link between these extracellular components and the actin cytoskeleton is thought to be part of the molecular pathogenesis of the muscular dystrophy phenotype in LGMD2I.

Muscular dystrophies are characterized by muscle wasting and repeated cycles of muscle fiber necrosis and regeneration. In mature muscle, the regenerative capacity depends on a pool of quiescent satellite cells, which are activated in response to injury, migrate and fuse with damaged myofibers. Hence, ongoing need for muscle fiber maintenance requires a continuous supply of functional satellite cells throughout life. Protein turnover is elevated in patients with muscular dystrophy, but the loss of muscle mass must inevitably mean that protein degradation within the myofibers, in the long run, outmatches synthesis.

A prominent proximal muscle weakness and atrophy at onset is observed in patients with LGMD2I. The progressive muscle wasting is often regional in muscular dystrophies, and the underlying mechanisms behind this variability in disease severity among muscles are poorly understood. We have recently studied muscle regeneration in a group of LGMD2I patients and found that there is a significant difference in ongoing or acute regeneration, which is substantially higher in patients who are compound heterozygous versus homozygous for the p.L276I mutation. This degeneration-regeneration cycle has an impact on the fiber type and size variability, which increases with disease severity. In postnatal healthy muscle, the mean fiber size does not differ more than 12% between fiber types [3]. If the fiber type specific atrophy and hypertrophy factors (number of very small or large fiber diameters multiplied by 1000 divided by the number of all fibers between 40–80 µm) exceeds 150, (or a hypertrophy factor above 400 for type 2 fibers) the muscle is considered pathological [3].

In the present study, we have studied some of the key intracellular signaling pathways, the regenerative response to determine if any changes in these could contribute to some of the clinical features observed in the muscles of patients with LGMD2I. We have simultaneously sampled a proximal and a distal muscle in each individual, in a group of patients with LGMD2I and in healthy subjects in order not only to determine any difference between healthy and affected individuals, but also to demonstrate changes in signaling and regeneration between the affected proximal and much less affected distal muscles. This simultaneous sampling is necessary to reduce fluctuations in signaling pathways due to differences in nutritional status.

## Results

### MRI and Isometric Force

**Figure 1:**
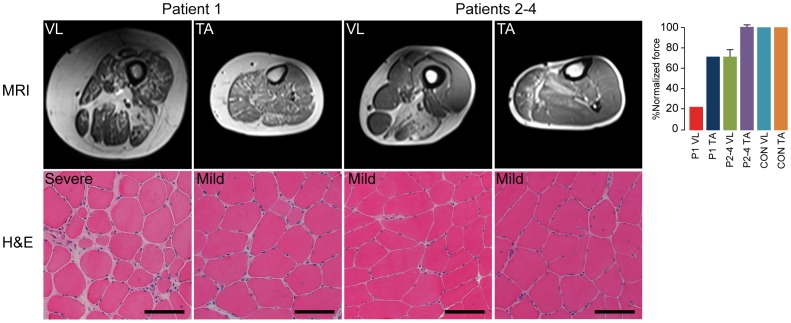
MRI and H&E stain of the biopsied vastus lateralis and tibialis anterior muscles. Biopsies were graded mild to severe based on the dystrophic changes as fiber size variability, level of degeneration and level of fibrosis as described previously [14], scale bar = 100 µm. Normalized muscle force of patients (P) to healthy control (CON) values, mean knee extension for VL and dorsal flexion for TA.

The MRI demonstrated severe dystrophic changes in the vastus lateralis muscle and to a lesser degree in the anterior compartment of the lower leg in patient 1. In patients 2–4, both tibialis anterior and vastus lateralis muscles were mildly affected (figure 1). The normalized force of vastus lateralis of patient 1 compared to healthy controls was clearly diminished at 24% of normal, while the normalized force of tibialis anterior was at 72%. The normalized force of vastus lateralis in patients 2–4 was 72±7% and 101±2% for tibialis anterior (figure 1).

### Morphology

**Figure 2:**
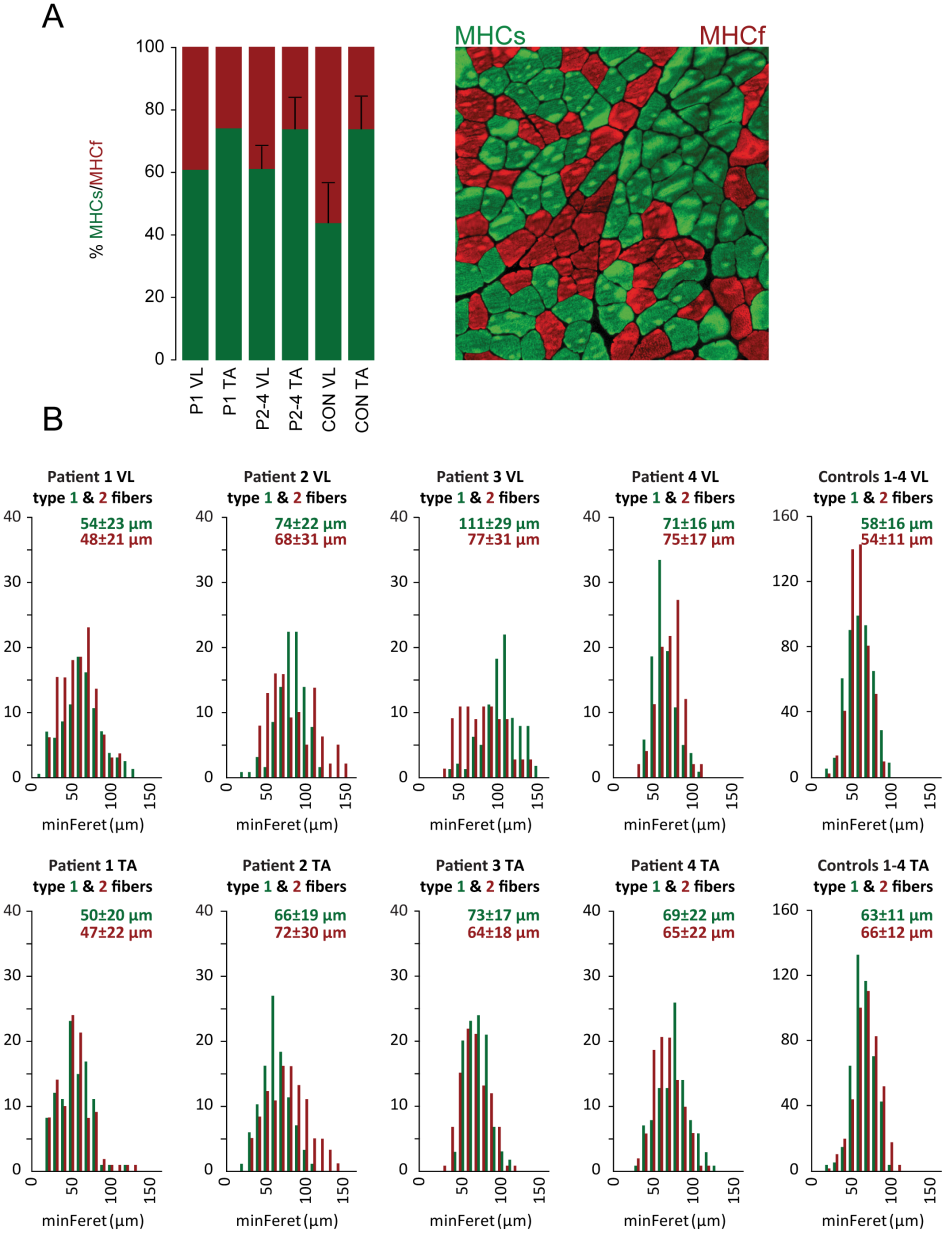
Immunohistology of muscle sections. A. Myofiber composition of patients (P) to healthy control (CON) values detected by immunohistochemistry. B. Fiber size distribution of type 1 (green) and type 2 (red) fibers in vastus lateralis (top row) and tibialis anterior (bottom row) in patients and controls. Data is based on 100 fibers randomly chosen fibers of each fibertype in each patient and 400 fibers of each type in the controls. Fibers are grouped into bins of 10 µm. minimal Ferets diameter Mean ± SD is given above each histogram.

The H&E staining of the biopsies clearly demonstrated fibrosis, internally nucleated fibers, cellular infiltration and irregular fiber size in the vastus lateralis of patient 1, while the tibialis was much less affected with internally nucleated fibers and minor cellular infiltration. The vastus lateralis and tibialis anterior muscles of patients 2–4 were similar and demonstrated internally nucleated fibers and irregular fiber size and shape, but no fibrosis. Myofiber composition showed a shift towards more type 1 fibers in the vastus lateralis of patients (61±6%) compared to the controls (44±13%) (figure 2A). In tibialis anterior, there was no difference in myofiber composition between patients and controls. Patient 1′s vastus muscle showed an increased atrophy factor for both type 1 (342) and 2 (389) fibers compared to the upper limit of 150 as well as increased hypertrophy factor (243) for type 1 fibers. No other patient or control had atrophy factors above the upper limit. However, the muscles of patient 2–4 had hypertrophy levels above the upper limit (figure 2B, table 1).

**Table 1:** Morphometric findings in patients with Limb girdle muscular dystrophy (LGMD2I) and healthy controls.

	Atrophy factor		Hypertrophy factor	
Subject	Type 1	Type 2	Type 1	Type 2
Patient 1 VL	342*	389*	247*	347
*Patient 1 TA*	*364*	*91*	*417*	*222*
Patient 2 VL	59	125	1627*	1500*
*Patient 2 TA*	*113*	*79*	*282*	*1444*
Patient 3 VL	0	20	23818*	2580*
*Patient 3 TA*	*0*	*13*	*559*	*423*
Patient 4 VL	0	0	304*	966*
*Patient 4 TA*	*15*	*26*	*970*	*372*
Control 1–4 VL	55	43	112	128
*Control 1–4 TA*	*33*	*47*	*21*	*324*

Asterisks (*) mark values above the upper limit for atrophy and hypertrophy factors. Upper limits for the value of atrophy factor is 150 both for type 1 and 2 fibers in vastus lateralis. Upper limits for hypertrophy factor is 150 for type 1 fibers and 400 for type 2 fibers in vastus lateralis. Values for tibialis anterior are shown in italics and included for comparison.

### Regeneration

**Figure 3:**
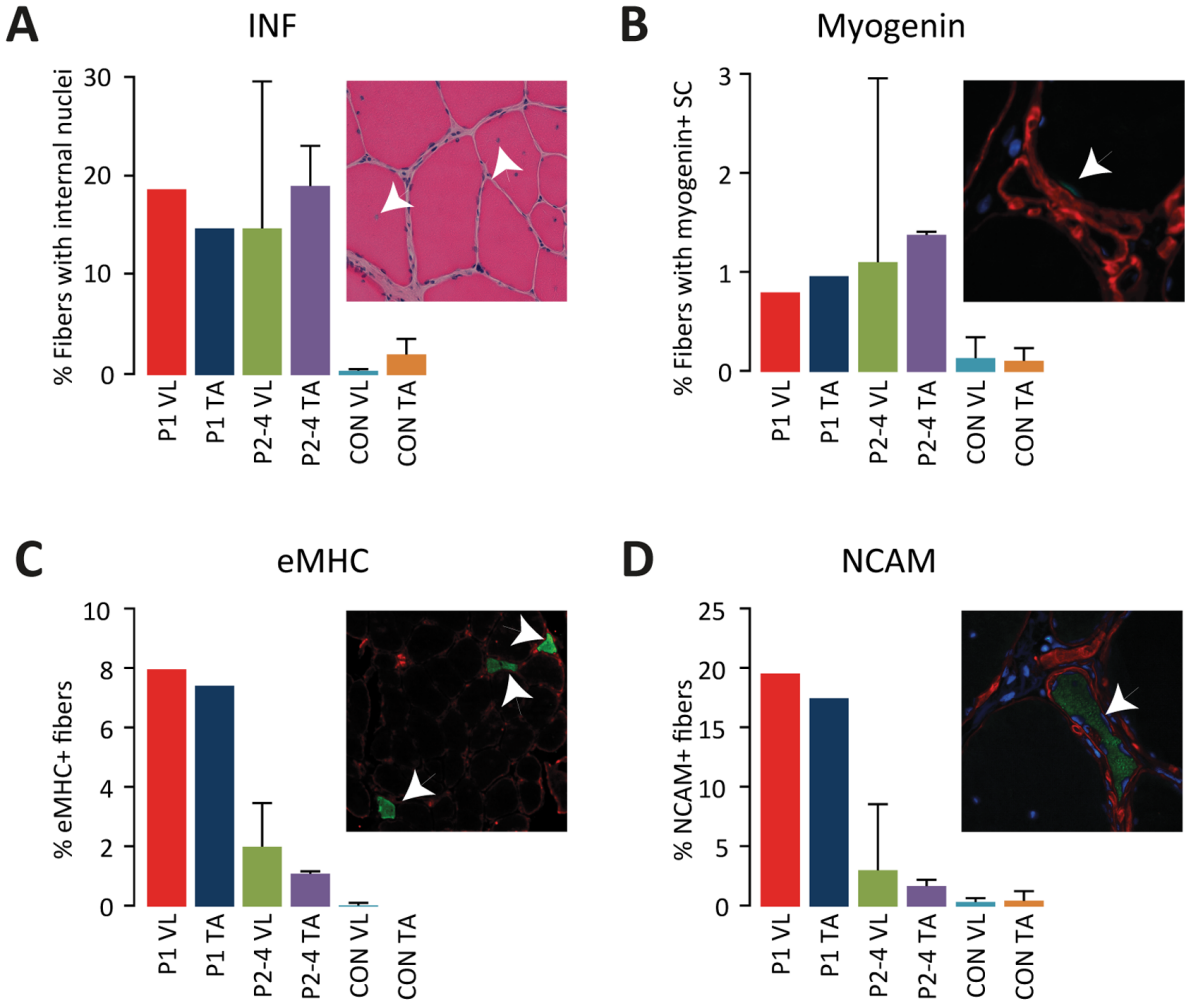
Markers for regeneration A. Proportion of fibers with internal nuclei. B. Proportion of fibers with satellite cells positive for myogenin. Proportion of actively regenerating fibers, eMHC (C) and NCAM (D). All error bars shown are standard deviations.

Fibers with internal nuclei were higher in patients with LGMD2I (18±8%) compared to healthy controls (1±1%), but no difference was found between proximal and distal muscles among the patients (figure 3A). We were not able to determine any myogenic transcription factor MyoD positive nuclei in the muscles of the included patients, while the level of myogenin positive satellite cells in the muscles from affected patients were elevated compared to controls (figure 3B). Actively regenerating fibers as determined by eMHC and NCAM expression, were highest in patient 1 and equally abundant in vastus lateralis and tibialis anterior, and elevated in patients 2–4 compared to controls (figure 3C and 3D).

### Western Blot

**Figure 4:**
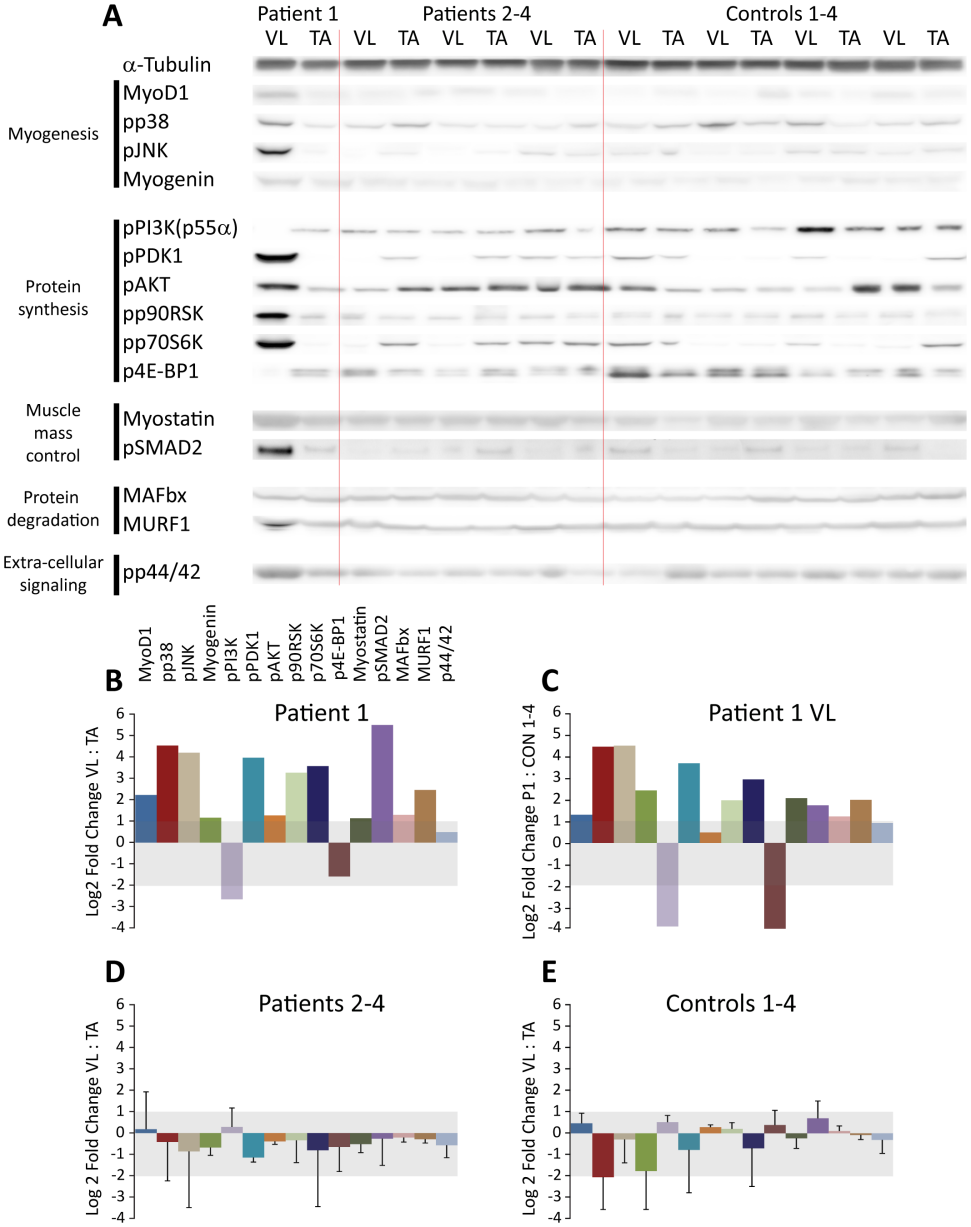
Western blot analysis. A. Western blot, the raw data used for calculating protein expression measured by immunoreactive bands on western blot. B, D, E. Bar charts show Log2 expression ratios of vastus lateralis and tibialis anterior normalized to loading control (alpha-Tubulin) for patient 1 (B), Patients 2–4 (D) and controls 1–4 (E). Shaded area signifies that the ratio or range is between 0.5 (Log2 = -2) and 2.0 (Log2 = 1). All error bars shown are the standard deviations. C. Log2 expression ratios of vastus lateralis of patient 1 and the healthy controls 1–4.

Our results show an overall increase in the level of expression/activation of the factors that we have investigated in the vastus lateralis muscle of patient 1 (figure 4A). Specifically, MyoD and myogenin expression and the active form of p38, potentially involved in myogenic signaling, was increased in the vastus lateralis muscle of patient 1 (figure 4B–D). The vastus muscle of this patient had marked alterations of investigated components of the protein synthesis as activation of the negative regulator PI3K (p55α) of protein synthesis pathway was decreased, activation of PDK1, Akt and p70S6K was increased combined with decreased activation of 4E-BP1. Myostatin, a negative growth control factor, and protein breakdown markers MAFbx and MURF1 were upregulated, thus protein turnover, both synthesis and breakdown, were highly increased in severely affected muscles. Activation of cellular stress markers p90RSK, p38 and JNK and the mitogen-activated protein kinase (MAPK) p42/44 were increased as well.

## Discussion

This case study provides new information about muscle regeneration and molecular response in mildly and severely affected muscles in patients with LGMD2I. While immunohistology did not demonstrate any difference in overall regeneration between severely and mildly affected muscles, we noticed a substantially higher ongoing regeneration in both muscles of the severely affected patient compared to the mildly affected patients. Second, western blotting revealed a marked increase in activity of key molecular signaling pathways, specifically myogenesis, protein synthesis and degradation in the more severely affected proximal compared to distal muscle. Third, the affected proximal muscles do adapt to the continuous degeneration by changing the fiber type composition towards more type 1 fibers.

In this study, the muscle of the severely affected patient demonstrates that histology/immunohistology may not reveal how complex molecular processes ultimately affect the progression of the disease. In the severely affected muscles, an approximately 6-fold increase in MyoD, a transcription factor for initiation of myogenesis and myogenin, expressed during terminal differentiation and fusion of myoblasts, suggests an increased activation of satellite cells, likely due to the increased myofiber damage in these muscles. Consistent with the upregulated myogenic factors, we find that severely affected muscles had a highly increased activity of the protein synthesis and ERK/p90RSK growth and differentiation pathways. While this is presumably part of the repair of the myofibrillar structures and should be considered beneficial to the muscle, unfortunately protein degradation and myostatin, was also increased in severely affected muscle as well. The increased activity of myogenic factors in severely affected muscles may lead to an adverse activation of myostatin as recent studies have demonstrated that an increase in MyoD1 may trigger the myostatin pathway for growth control as part of a negative feed-back mechanism [4], in addition myostatin is able to suppress MyoD expression so not only atrophy is induced but myogenesis is effectively down-regulated [5]. Surprisingly, immunohistological detection of eMHC and NCAM did not demonstrate any difference between severely and mildly affected muscles in the severely affected patient as the dramatic increase in MyoD expression would otherwise suggest. As these are considered markers for ongoing regeneration, it is possible that the regeneration process in the proximal muscle is running at maximum. However, this is insufficient to counter the ongoing degeneration, likely for a number of reasons of which premature activation of the myostatin-initiated negative growth control could be one. The upregulation of MuRF1 and MAFbx, ubiquitin ligases and downstream effectors of myostatin, may destabilize the myofibrillar structure as MuRF1 can bind to the C-terminal part of the giant sarcomeric protein titin and possibly modulate titin signaling [6] and myosin heavy chain content [7] while MAFbx appears to act on signaling affecting myogenesis and differentiation as MyoD, myogenin and the transcription initiation factor eIF3-f are targets for MAFbx [8], [9].

This mechanism is finely tuned in normal individuals ensuring that the muscle mass is appropriate. However, in patients with LGMD2I this part of the mechanism could be skewed, which due to the targets involved not only affects protein synthesis, but also terminal differentiation and fusion. The highly increased activity of the stress kinases p38 and JNK may be explained by the increased satellite cell activation and presumably the continuous pathological state of the muscles, as p38 has been demonstrated to be a direct activator of myogenic factors along with MyoD, leading to transcription of muscle specific genes, among them myogenin [10], [11]. JNK has been demonstrated to be necessary for proper differentiation of myoblasts, however, overexpression of a key JNK effector, the c-Jun transcription factor, results in repression of MyoD expression to a basal level suggesting that JNK regulation of myogenesis may be part of a feed-back mechanism [12], [13].

The mechanism of formation of large and small diameter is not fully understood. Small fibers can be a product of degenerated fibers or lack of functional regeneration or branching of the myofiber. Large fibers can also be a result of correct regeneration with abnormal cytoarchitecture. The abnormal atrophy factor for both fiber types in vastus lateralis of patient 1 may be explained with the increased muscle regeneration in this patient, generating multiple new fibers. The increase in hypertrophy factor for type 1 fibers in the vastus muscle of patient 1 could be caused by compensatory hypertrophy. Some conversion of type 2 to type 1 fibers and fiber size variability is seen in many classic muscular dystrophies. The absence of any increased atrophy factor above the upper limit for patient 2–4 is consistent with the histopathology, where hardly any small regenerating or atrophic fibers are seen. It appears that these mildly affected patients compensate for the disorder by moderate fiber hypertrophy.

This study demonstrates some of differences between severely and mildly affected muscles that otherwise go unnoticed if muscles were evaluated by histology alone. Whether the dramatic increase in protein turnover in proximal muscles of severely affected patients can be avoided by exercising the muscles is difficult to say. Less use may reduce the wear and tear of the muscles, but may also lead to a lot of other problems like disuse-related atrophy and risk of diabetes. More use of the muscles may increase the wear, but may also initiate regeneration related structural enhancement of the muscle fibers in the form of increased levels of supporting membrane proteins, i.e. α7/β1 integrin. While this case study primarily demonstrates the difference in cellular signaling between a severely affected patient and mildly affected patients it also reveals some potential targets of therapeutic intervention as select signaling inhibitors may decrease the detrimental effect of excess protein degradation, one obvious being a myostatin inhibitor due to myostatin’s potent influence on myogenesis and protein degradation. These data are significant in identifying therapeutic targets specific to LGMD2I.

## Materials and Methods

### Ethics Statement

All investigations were performed with written informed consent and in accordance with the Declaration of Helsinki, and the study was approved by the Danish Committee System on Biomedical Research Ethics ID: H-1-2010-021.

### MRI, Biopsies and Manual Muscle Testing

Four patients with LGMD2I homozygous for p.L276I (age 48±19 years) and four sedentary age- and gender-matched controls (age 42±15 years) participated in this study. Leg muscles of patients were imaged by MRI, and the ankle dorsal flexion and knee extension forces, corresponding to the muscle that was biopsied was measured using hand-held dynamometry. Muscle force measurements were normalized to forces found in healthy subjects. Guided by MRI, percutaneous needle muscle biopsies were obtained from a proximal (vastus lateralis) and a distal muscle (tibialis anterior) in all subjects, which were snap-frozen in liquid nitrogen-cooled isopentane, and stored at -80°C.

### Histology

Histological and immunohistological analyses were performed to clarify the morphological alterations and correlate these with the clinical findings. Five serial sections (10 µm thick) were fixed in acetone and methanol (1∶1). The first section was stained with H&E to count fibers with internal nuclei (defined as non-peripheral) and to grade biopsies based on the dystrophic changes as fiber size variability, level of degeneration and level of fibrosis as described previously [14], [15]. The other four sections were blocked in buffer (3% fetal calf serum in PBS) prior to staining. Primary antibodies were diluted 1∶100 and incubated overnight. We used A4.74 and BA-D5 antibodies for fast and slow fibers (DSHB, Iowa City, IA). Embryonic myosin heavy chain (eMHC) (F1.652, DSHB, Iowa City, IA) and neural cell adhesion molecule (NCAM/CD56, Novocastra, UK) to assess actively regenerating myofibers, so we excluded muscle fibers showing an intermediate eMHC or NCAM stain and nuclear clumps (with positive staining). Differentiating satellite cells were visualized staining for myogenin (clone F5D, DSHB, Iowa City, IA), DAPI nuclear stain (Invitrogen, Carlsbad, CA) and to be in a satellite cell position using laminin (L9393; Sigma, St Louis, MO). Alexa 488 and 594 (Invitrogen, Carlsbad, CA) secondary anti- mouse and anti-goat antibodies were used at a 1∶500 dilution in PBS buffer. For determination of positive fibers for regeneration markers, entire sections with of a range of 364 to 1005 fibers were counted. For each muscle sample, the minimal Feret diameter was measured in 100 fibers of each fiber type using ImageJ v.1.41 [16]. The sections were observed under a Nikon Eclipse 80i microscope with epi-fluorescence. Atrophy factor express the proportion of fibers below a diameter of 40µm of the number of fibers with a diameter between 40 and 80 µm (normal range of fiber diameter) after the fiber diameters have been binned in 10 µm increments for histogram analysis. The hypertrophy factor similarly express the proportion of fibers above a diameter of 80 µm. Calculation of atrophy and hypertrophy factors were carried out according to Brooke & Engel [3]. Since the upper limits have been described for vastus lateralis only, we have in addition to the numbers for vastus included the numbers for tibialis anterior for comparison only and not made any assumptions as to whether these are below or above a yet undefined upper limit.

### Western Blotting

Muscle was homogenized using a bead-mill at 4°C in ice-cold lysis buffer (10 mM Tris,5 mM β-glycerophosphate, 1.15 mM Na_2_MoO_4_, 1 mM Na_3_VO_4_,4 mM Sodium tartrate, 1 mM EGTA, 1 mM NaF, 2 mM Imidazole, 2 mM Na_4_P_2_O_7_, 0.1% Triton-X 100, 1 mM EDTA, 0.5% Sodium Deoxycholate,1 mM DTT, 20 µM Leupeptin, 20 µM Pepstatin, 0.07 U/ml Aprotinin, 1 mM PMSF, 10 nM Calyculin, 5 µM Cantharidin). Supernatants were collected and protein concentrations were determined using the Bradford assay. Equal amounts of extracted muscle proteins were separated on 10% TGX polyacrylamide gels (Bio-Rad, Hercules, CA) at 200V for 30 min. Proteins were transferred to PVDF membranes and post transfer membranes were stained with Sypro Ruby (Sigma-Aldrich, St Louis, MO) to ensure equal protein transfer. Membranes were blocked in Baileys Irish Cream (Dublin, Ireland) for 30 min and incubated overnight with primary antibodies Alpha-Tubulin (12G10) and Myogenin (clone F5D, DSHB, Iowa City, IA); MyoD1 (clone 5.8A, VP- M669, Vector Laboratories, Burlingame, CA); Myostatin (AB3239, Millipore, Billerica, MA); MAFbx (SAB2501208) and MuRF1 (SAB2105510) from Sigma-Aldrich, St Louis, MO; p38, phospho-Thr180&Tyr182 (#4631)/PI3K, phospho-Tyr458(p85)/Tyr199(p55) (#4228)/PDK1, phospho-Ser241 (#3061)/Akt, phospho-Ser473 (#4060)/p70S6K, phospho-Thr389 (#9205)/4E-BP1, phospho-Thr37/46 (#9459)/Smad2, phospho-Ser245/250/255 (#3104) all from Cell Signaling Technologies, Danvers, MA. Secondary antibodies coupled with horseradish peroxidase diluted 1∶10000 were used to detect primary antibodies (DAKO, Denmark). Immuno-reactive bands were detected using SuperSignal West Dura kit (Thermo Scientific, Waltham, MA), quantified using a Gbox XT16 darkroom and GeneTools software (Syngene, UK) was used to measure the intensities of the bands on 16-bit digital photos. Alpha-tubulin was quantified on one blot and used as a loading control to normalize all the other targets.
